# Oxytocin Receptor Gene Polymorphisms and Early Parental Bonding Interact in Shaping Instagram Social Behavior

**DOI:** 10.3390/ijerph17197232

**Published:** 2020-10-03

**Authors:** Andrea Bonassi, Ilaria Cataldo, Giulio Gabrieli, Jia N. Foo, Bruno Lepri, Gianluca Esposito

**Affiliations:** 1 Department of Psychology and Cognitive Science, University of Trento, 38068 Rovereto, Italy; andrea.bonassi@unitn.it (A.B.); ilaria.cataldo@unitn.it (I.C.); 2 Mobile and Social Computing Lab, Bruno Kessler Foundation, 38122 Trento, Italy; lepri@fbk.eu; 3 Psychology Program, School of Social Sciences, Nanyang Technological University, Singapore 639818, Singapore; GIULIO001@e.ntu.edu.sg; 4 Human Genetics, Genome Institute of Singapore, Singapore 138672, Singapore; jianee.foo@ntu.edu.sg; 5 Lee Kong Chian School of Medicine, Nanyang Technological University, Singapore 308222, Singapore

**Keywords:** gene*environment, parental care, parental overprotection, oxytocin receptor gene, rs2254298, rs53576, online behavior, Instagram, social network

## Abstract

Human beings engage in multiple social interactions daily, both in person and online. There are, however, individual differences in the frequency and quality of these interactions. This exploratory study focuses on online interactions and aims to model these differences by looking at potential environmental and genetic factors. The environmental factor is the childhood parental relationship, as reported by the participants in the dimensions of the Parental Bonding Instrument (*N* = 57, 41 females). At a genetic level, buccal mucosa cell samples were collected to assess participants’ genetic susceptibility, and OXTr regions rs2254298 (G/G homozygotes vs. A-carriers) and rs53576 (A/A homozygotes vs. G-carriers) were analyzed. To capture participants’ online activity, Instagram was probed. The number of people that the individual follows (“followings”), followers, and posts were used as a proxy for the quantity of interaction, and a Social Desirability Index (SDI) was computed as the ratio of followers to followings. An interaction between OXTr groups and parental bonding scores on the number of followings and posts was hypothesized. A gene-environment interaction for OXTr/rs2254298 on the number of Instagram posts was identified. In line with the hypothesis, participants with a genetic risk factor (A-carriers) and a history of low paternal care showed fewer Instagram posts than those without this risk factor (G/G genotype). Moreover, an interaction effect between maternal overprotection and OXTr/rs2254298 on the Instagram SDI was detected. These findings could represent an indirect pathway through which genes and parental behavior interact to shape social interactions on Instagram.

## 1. Introduction

Humans are inherently social creatures. They cooperate to satisfy physiological and affiliative needs in terms of care, protection, and reproduction [1], thus safeguarding their survival from environmental hazards [2] and increasing their sense of inclusiveness [3].

From the first years of life, infants are exposed to a stimulating environment that strengthens their social bonds with parents and family members [4]. During infancy and childhood, the individual lays the basis for a complex pattern of exchange and engagement through his/her interaction with relatives and peers [5]. The distinctive pattern in early attachment with caregivers can be stable across human development and influence adult attachment with partners [6,7]. Within the interaction between caregivers and offspring [8], high quality parental bonding enhances children’s self-efficacy [9], as well as social and emotional communication [10], while reducing psychological [11] and physiological [12,13] stress. Parental bonding represents the core causal factor of the main developmental stages, which remain open to modulation due to experience and exposure to further environmental events [14].

Currently, both digital and physical spaces can forge social bonds and become the theaters of online and offline interactions that affect relationships among people. The adoption of technological devices allows children to explore social media platforms and online worlds [15]. Hence, the growing social skills in virtual and real environments shape adolescents’ [16,17,18] and adults’ online and offline behaviors [19,20,21]. In particular, the ubiquitous usage of Social Network Sites (SNSs) has created a virtual environment where social interactions can happen anytime and anywhere [22,23,24,25,26]. Among the different platforms, Instagram, one of the most popular sites for youths [27], focuses on photo-sharing and visual content. It is mainly characterized by various online social behaviors such as scrolling through followings’ contents, watching stories, publishing posts, tagging followers, commenting on photos, and chatting privately [28].

With the advent of Instagram, kids are early participants of social media. Parents become prey to “sharenting”, the phenomenon that describes the tendency to exhibit pictures of their children online [29]. Interestingly, recent findings have highlighted a disagreement regarding the positive and negative outcomes of online social interactions on SNSs. For instance, the frequency of Facebook and Instagram usage, as well as high rates in reported self-attractiveness [30] are associated not only with a higher self-esteem [31], but also with depressive symptoms [32]. Furthermore, Instagram improves happiness and decreases solitude with the intimacy offered by the images [33]. At the same time, an increased number of Instagram posting activities, combined with individual body dissatisfaction, increases the probability of engaging in negative romantic relationships [34]. The reason for Instagram use was also traced to a high need for interpersonal interaction and dominant traits in narcissism [35,36]. The same parents share their “family snapshots” to prove that the “merry family ideal” and the “cute child ideal” have come true [37]. Although several papers investigated how parental factors are associated with user activity on Facebook [38,39,40] and Instagram [29,37,41], there is a lack of research focused on the interplay between Instagram activity and early attachment with parents. This gap is even more evident if the potential relationship between attachment and genetic factors is considered. Only a few studies recently probed the genetic influence on the frequency of social media use [42,43].

The evolutionary tendency to be social can be modulated by genetic factors, which, in turn, are regulated by the environmental actions across the human lifespan, thus conferring nuanced levels of sensitivity to the experiences. Based on the model of the plasticity of genes [44,45], it is not only the protective factor versus the risk factor of genes that determines human behavior. Indeed, one must also take into consideration the quality of environmental factors that interact with allelic expression in shaping physiological responses and then behavioral patterns that foster environmental adaptation [46].

According to the susceptibility hypothesis or sensitivity hypothesis [47], alleles (i.e., G or A) of a given genetic region (i.e., oxytocin receptor gene rs2254298) are associated with different degrees of sensitivity to the environmental effects (i.e., quality of early parental care) [48]. Here, a genetic risk factor makes the individual genetically sensitive or susceptible to life events and individual experience [49,50].

In the context of sociability, people with high genetic sensitivity will exhibit a more adaptive social behavior when exposed to a positive environment (i.e., a warm relationship with parents) [51,52]. However, they will display less adaptive social behavior if they go through negative events (i.e., the loss of a parent, child abuse, or maltreatment) [53,54]. Conversely, low genetic sensitivity and vulnerability will make someone more resistant to the effects of early traumatic episodes (i.e., parental separation or abandonment), as well as beneficial events (i.e., competent caregiving), generating a decreased social response [55].

Within this debate, multiple studies have attributed a key role in the biological explanation of social behavior to the hormone oxytocin [56,57,58]. Specifically, rs53576 and rs2254298 polymorphisms, encoded by the Oxytocin receptor gene (OXTr), correlate with social behaviors, social cognition, and empathy [59,60]. For each region, two allelic structures have been observed to play a role as determinants in social development: guanine (G) to adenine (A) substitution shows greater sensitivity to the environment and influences responses to stressful life events [61]. However, from the previous results, it is not possible to unequivocally establish which variation—G or A—is more associated with less adaptive social responses.

Concerning rs2254298, a history of paternal overprotection was found to moderate the heart rate responses to socially distressing stimuli (increased for A-carriers, but decreased for G/G homozygotes) [62]. A-carriers also showed lower empathetic levels than G-carriers regardless of parental warmth [60].

As for rs53576, individuals with the G variation show a variety of favorable features when compared to A-carriers, such as higher levels of trust [63], dispositional empathy [64], greater sympathetic response to stressors [50], and more sensitivity to social cues [65] and social interactions [66]. Moreover, social support before a distressing task was observed to reduce cortisol response in G-carriers, but not in A/A homozygotes [67].

Taken together, these scientific contributions point out that genetic expression, combined with early environmental exposure, contribute to the shaping of adult sociability. However, the role played by specific genetic predispositions (i.e., the ones related to early environment exposure during infant-parent interaction) on the online social relationships of adults is considerably underexplored.

This study investigates how OXTr and caregivers’ propensities during childhood interact in modulating adult online relationships on Instagram (see Appendix A). Specifically, two OXTr Single-Nucleotide Polymorphisms (OXTr SNPs: rs2254298 and rs53576) for the genetic component and the Parental Bonding Instrument as the assessment for the parent-child recalled bonding were considered. Likewise, the Instagram number of (i) followed users (here called “followings”), (ii) published posts, (iii) followers, and (iv) a further combined index, called the “Social Desirability Index” (SDI), were selected as the main social media variables.

This goal was addressed with the formulation of a combined directional hypothesis to evaluate both correlations and mean level similarities and differences between conditions. In favor of the sensitivity hypotheses, for each Instagram variable, an interaction effect between the genetic component and the parental attachment scores, independent of gender, was hypothesized. More specifically, adult Instagram users with a genetic risk factor (OXTr rs2254298 A-carriers, OXTr rs53576 G-carriers) and who were exposed to a beneficial and positive early relationship with their parents (high parental care, low parental overprotection) would show increased online social activity (higher number of posts and followings) compared to less vulnerable genetic carriers (OXTr rs2254298 G/G homozygotes, OXTr rs53576 A/A homozygotes). Conversely, Instagram users with a genetic risk factor and who were exposed to an adverse and negative early relationship with parents (low parental care, high parental overprotection) would show decreased online social activity as described by a lower number of posts and followings compared to less vulnerable genetic carriers.

## 2. Material and Methods

### 2.1. Participants

Sixty-one (*N* = 61) non-parent Singaporean adults were recruited among the students of the Nanyang Technological University. Exclusion criteria were: (i) current or lifetime history of genetic, neurological, or psychiatric disorders, (ii) age higher than 30 years old, and (iii) being a parent. Inclusion criteria were: (i) owning an Instagram account; (ii) using Instagram at least once a week. Four participants were not included due to technical issues; thus, the final sample consisted of 57 Singaporean adults (16 males and 41 females) aged 18–25 years old (*M* = 20.82, *SD* = 1.59) (see Appendix B).

### 2.2. Procedure

Participants were recruited among students of the Nanyang Technological University and rewarded with academic credits for their participation. Prior to data collection, participants signed an informed consent form and provided demographic information. Then, using the web-based survey platform Qualtrics, participants filled in a self-reported questionnaire (i.e., the Parental Bonding Instrument) to assess their recalled parental bonding during childhood. In addition, a sample of buccal mucosa was collected from each participant by using sterile cotton swabs (Medline MDS202010Z Sterile Cotton Tipped Applicator, 6”) and sent to a laboratory for genotyping. Lastly, participants provided the link to their Instagram profile, and using a Python program, four indicators of their Instagram activity were automatically extracted. Where the Python program failed in extracting the data, Instagram information was collected manually. The four indicators of Instagram activity, later elaborated, were publicly available regardless of the privacy settings of each user’s account. This research was approved by the Ethical Committee of Nanyang Technological University (IRB-2015-08-020-01) (see Appendix C).

### 2.3. Parental Bonding

The Parental Bonding Instrument (PBI) [68] is a 50 item self-reported questionnaire widely adopted to measure individual self-perception of attachment with their parents during the age of 0–16. In this questionnaire, participants reported the quality of the parental bonding, as well as the caregiving behavior they experienced during childhood and adolescence on a Likert scale from 0 (“very unlike”) to 3 (“very like”). The PBI (average Cronbach’s α=0.88) investigates two main dimensions of recalled care and overprotection of both parents, calculated by summing the items and considering the scores of six specific items as reversed for each dimension (Table 1). The questionnaire is then divided into four subscales: paternal care (12 items), maternal care (12 items), paternal overprotection (13 items), and maternal overprotection (13 items). Parental care (e.g., “Appeared to understand my problems and worries”) is a measure of affection, warmth, emotional affinity, and empathy, and its scoring is positively correlated with the quality of the parental bonding [69]: the higher the score, the higher the level of affection perceived during the past early parent-child interactions. On the other hand, parental overprotection (e.g., “Tried to control everything I did”) is a measure of the level of control, intrusion, and restriction from autonomy, and its scoring is negatively correlated with the quality of the parental bonding [70]: the higher the score, the higher the level of oppression perceived during the past early parent-child interactions.

### 2.4. Genetic Assessment

The same data extraction procedure used by Bonassi et al. [71] was adopted in this study. Specifically, DNA derivation and genotyping were executed by ACGT, Inc. (Wheeling, IL, USA). DNA was extracted from each kit using the Oragene DNA purifying reagent, and DNA concentrations were assessed through spectroscopy (NanoDrop Technologies, Wilmington, NC, USA). Each DNA sample was magnified through Polymerase Chain Reaction (PCR) for the OXTr gene rs2254298 region target with the primers 5-TGA AAG CAG AGG TTG TGT GGA CAG G-3 and 5-AAC GCC CAC CCC AGT TTC TTC-3. A PCR reaction of 20 llcomprising 1.5 ll of genomic DNA from the test sample, PCR buffer, 1 mM each of the forward and reverse primers, 10 mM deoxyribonucleotides, KapaTaq polymerase, and 50 mM MgCl2 was conducted. The PCR process consisted first of a 15 min denaturation at 95 °C, 35 cycles at 94 °C (30 s), 60 °C (60 s), and 72 °C (60 s), and a final 10 min protraction at 72 °C. PCR reactions were genotyped with an ABI 3730xl Genetic Analyzer (Applied Biosystems Inc., Zug, canton of Zug, Switzerland) and standardized with GeneScan 600 LIZ (Applied Biosystems, Inc.) size standards on each sample. Genotypic data were examined using GeneMapper ID (Applied Biosystems, Inc.).

For this DNA region, participants having at least one A allele (A/A homozygotes or G/A) were classified into a single A-carriers group. The average distribution of the different genotypes in the Asiatic population is 61% for G/G homozygotes and 39% for A-carriers (1000 Genomes project, BioSamples: SAMN07486027-SAMN07486024, dbSNP (Short Genetic Variations), 2017), whereas the distribution in this sample was 58% for G/G homozygous and 42% for A-carriers. Genotype frequencies were as follows: A/A = 4 (7.02%), G/A = 20 (35.09%), G/G = 33 (57.90%). This genotype distribution follows the Hardy–Weinberg equilibrium ($X^{2}$ (1) = 0.16, ns). Participants’ age (*t*(55) = 0.64, *p* = 0.53) and gender ($X^{2}$(1) = 0.55, *p* = 0.46) did not significantly differ between the two groups G/G versus A.

Similar DNA procedures were applied to the OXTr gene rs53576 region target. However, the forward and reverse primers that were considered were instead 5-GCC CAC CAT GCT CTC CAC ATC-3 and 5-GCT GGA CTC AGG AGG AAT AGG GAC-3. For this DNA region, study participants having at least one G allele (G/G homozygotes or A/G) were classified into a single G-carriers group. The average distribution of the different genotypes in the Asiatic population is 30% for A/A homozygotes and 70% for G-carriers (1000 Genomes project, BioSamples: SAMN07486027-SAMN07486024, dbSNP (Short Genetic Variations), 2017), whereas the distribution in this sample was 35% for A/A homozygous and 65% for G-carriers. In detail, genotype frequencies were as follows: A/A = 20 (35.09%), A/G = 32 (56.14%), G/G = 5 (8.77%). This genotype distribution follows the Hardy–Weinberg equilibrium ($X^{2}$ (1) = 2.43, ns). Participants’ age (*t*(55) = 0.09, *p* = 0.93) and gender ($X^{2}$(1) = 0.55, *p* = 0.46) did not significantly differ between the two groups A/A versus G.

### 2.5. Instagram Variables

Four variables (i.e., number of followings, number of posts, number of followers, and the Social Desirability Index) were extracted from each participant’s Instagram profile. In the subsequent paragraphs, these variables are described in detail.

#### 2.5.1. Number of Followings

The number of followings is the number of followed profiles by a given participant. It describes the unidirectional extension of the social network from a given individual to other Instagram users. Participants with higher following numbers tend to invest more time in virtual social behaviors than the ones with few contacts [72]. Indeed, following more people means managing more social interactions [73], being exposed to more content, and consuming more activity in searching for other users or remaining updated with other users’ news [74]. This is particularly relevant if it is considered that Instagram users tend to compare themselves with others. People judge the way that the users who they follow behave or appear. This causative chain induces Instagram users to evaluate their own real-life and regulate their virtual activity by comparing it to the ones in their network.

#### 2.5.2. Number of Posts

The number of posts reflects the individual tendency to publish and share content and information on the personal profile. Differently from other SNSs, Instagram posts can only consist of pictures, and a brief caption of the pictures is optional. Previous research has shown that the number of posts is also positively associated with the users’ depressed mood [75] and the users’ body image [76]. Thus, these studies reported that intense online activity (i.e., publishing content) could be a marker of psychological vulnerability [77,78,79,80]. Moreover, the number of posts also describes: (a) the Instagram user not only as an observer, but also as a proactive and constructive agent of the social network, (b) the user’s availability to expose himself/herself to the others’ judgment for different personal reasons (e.g., openness, appearance, egocentricity, need for approval) and, at the same time, his/her aim at influencing others’ behavior [81], and finally, (c) the need to be pro-social and to connect with others by engaging with them through posts.

#### 2.5.3. Number of Followers

The number of followers underlines the multi-directional extension of the social network from other users to the assessed study participant. From a cognitive-behavioral view, followers’ approval operates as a positive or negative reinforcer of the user’s online behavior and as a moderator of the user’s social cognition. Therefore, the social network dictates the laws of online behavior, imposing and raising the ideal standard of virtual social interactions to which each single user should conform to increase his/her likeability [82]. The number of followers could be affected by the privacy level set by the user for his/her profile. Indeed, users with a private profile could get less followers than users with a public profile since a private profile can be followed only upon permission of the owner.

#### 2.5.4. Social Desirability Index

The Social Desirability Index is defined as the ratio between the number of followers and the number of followings, and it was estimated in order to investigate the asymmetry between these two quantities for each study participant’s network [83]. Interestingly, this ratio can disclose the tendency of some Instagram users to maximize the number of followers at the expense of the number of followings [84].

### 2.6. Statistical Analysis

The statistical analysis was conducted with R (Version 4.0.0). Each Instagram variable was standardized using z-scores (see Table 2).

Univariate and multivariate distributions of Instagram variables and attachment scores were examined for normality and the presence of outliers [85]. The distance of each observation to the centroid was estimated for outliers defined as having a value equal to 2 *SDs* above/below the mean. Out of the total of 57 observations for each Instagram variable, three extreme values were considered outliers for the number of followings and for the number of posts, two values for the SDI, and one value for the number of followers. Outliers were treated by means of winsorization [86], a method for treating outliers based on the weight modification of the extreme values. Thus, for each Instagram variable, this approach allowed the replacement of the outliers with the mean value in observations, obtained by excluding the outliers.

Then, for each Instagram variable, density and quantile-quantile plots were visualized, and the related skewness and kurtosis were computed (see Table 3). The obtained results were considered acceptable to prove a normal univariate distribution [87]. As for the number of posts, whose sampling did not show a Gaussian distribution, a logarithmic transformation was applied to assure the applicability of statistical parametric tests (see Table 3).

Additionally, the assumption of the homogeneity of variance and multicollinearity across the sample was ascertained (see Supplementary Materials).

A preliminary analysis of Instagram variables was conducted to exclude any effect that could be attributable to a difference in the distribution of participants’ gender (4 repeated measures, corrected α=0.0125).

Although hypothesis-driven analysis was fixed to the number of Instagram followings and posts, the analogous statistical procedure was adopted for exploratory analyses conducted on the number of Instagram followers and the SDI. Next, a differential Bonferroni correction was applied for the hypothesis-driven tests (2 repeated measures for each genetic variable, corrected α=0.025) and the exploratory tests (2 repeated measures for each genetic variable, corrected α=0.025). Throughout the data analysis, the genetic variables, OXTr rs2254298 and OXTr rs53576, were used as single predictors in separate analyses.

For each Instagram variable, one multiple regression was performed with the Instagram value as the dependent variable, the OXTr gene genotype rs2254298 (G/G and A-carriers) as a between-subjects factor, and all the PBI dimensions (i.e., maternal care, maternal overprotection, paternal care, and paternal overprotection) as continuous predictors. To appropriately test the hypotheses, for each Instagram variable, one linear regression was also performed with the OXTr gene genotype rs53576 (A/A and G-carriers) as a between-subjects factor, with the other parameters fixed and unvaried. For the overall Instagram-dependent variables, two main effects and two 2 way interaction effects related to OXTr rs2254298 were considered and represented by bar plots and scatterplots with linear models. Given that this study is focused on the role played by genetics in regulating Instagram social behavior, only the main effects of genotype and any significant interactions of a PBI dimension with genotype are discussed. The main effects of the PBI dimensions (i.e., those shown below) were included in the model only to support the plausible interpretation of data according to the gene*environment perspective. Pearson’s *r* and Fisher’s *z* [88] coefficients were also assessed to further investigate the effect of the continuous predictors on the dependent variable.

For each significant interaction effect between the PBI covariate and the genotype on the Instagram-dependent variable, the sample was divided into two groups (low vs. high PBI dimension) by the median split procedure (see Supplementary Materials). Post-hoc Student’s *t*-tests were computed within the low vs. high PBI groups to examine hypothetical significant differences between the two genetic carriers on Instagram behavior. *R* squared and Cohen’s *d* were estimated to evaluate the magnitude of the significant effects for linear models and Student’s *t*-tests, respectively. Post-hoc statistical power for linear multiple regression (fixed model, $R^{2}$ deviation from zero) [89] calculated with G*Power software (Version 3.1) is reported for each statistical test.

## 3. Results

### 3.1. Instagram Variables: Preliminary Results

**Figure 1:**
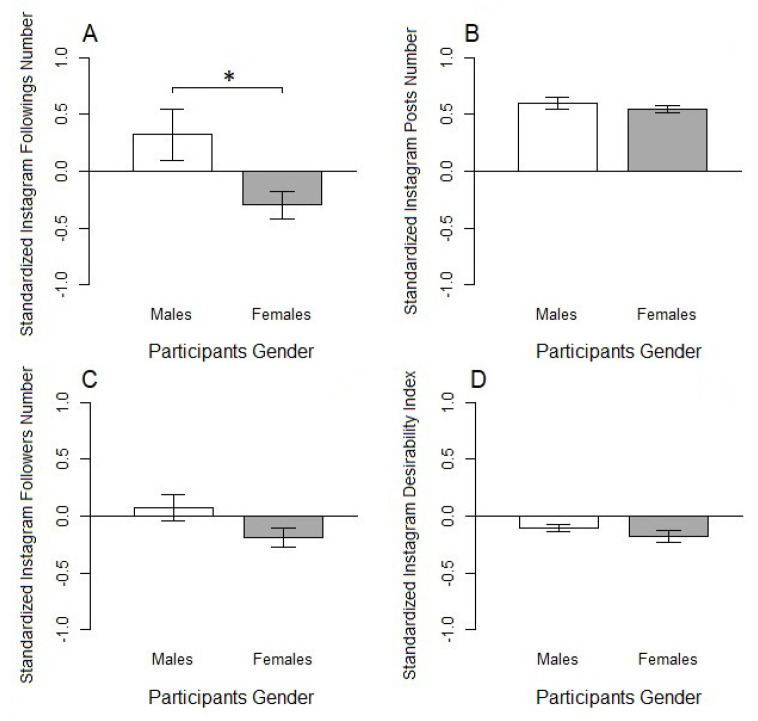
Effect of participants’ gender on each standardized Instagram variable. Bar plots are reported according the following order: (**A**) number of followings; (**B**) number of posts; (**C**) number of followers; (**D**) Social Desirability Index (* *p* < 0.0125).

Four preliminary two tailed Student’s *t*-tests were made to be sure that any significant effect on Instagram variables could not be attributed to participants’ gender (corrected α=0.0125). As expected, no significant differences in the standardized Instagram number of posts, followers, and the standardized SDI were found between male and female participants (Table 4). However, contrary to the expectations, the number of Instagram followings was higher in males than females (*t* = 2.60, df = 55, *p*< 0.012) (Figure 1). As a result, participant gender was included as a between-subjects variable on the number of Instagram followings.

### 3.2. Instagram Effects: OXTr rs2254298

#### 3.2.1. Number of Instagram Posts

The results of the regression analysis on the standardized number of Instagram posts indicated that paternal care was a significant covariate in both the main and the interaction effect with genotype ($R^{2}$ (95% CI [0.06, 0.47]) = 0.31, power = 0.95). Although a main effect of paternal care was found for the number of Instagram posts (β=0.02, SE = 0.01, *t* = 3.12, *p*= 0.003), the post-hoc two tailed Student’s *t* test revealed that the number of Instagram posts was not significantly different between the low vs. high paternal care groups (*t* = −0.71, df = 55, ns) (see Table 5).

**Figure 2:**
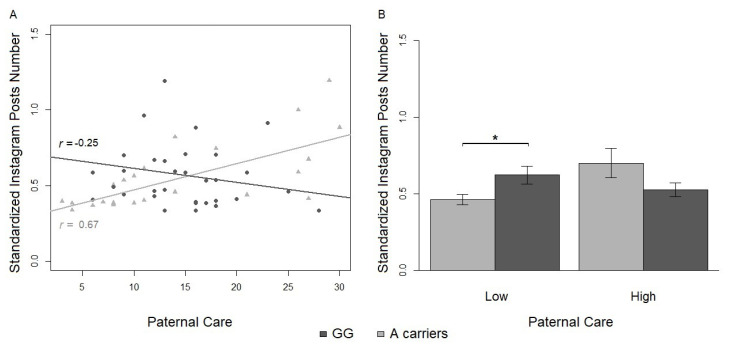
(**A**) Effect of the interaction between paternal care and genotype on the standardized number of Instagram posts. Correlations between the number of Instagram posts and the reported paternal care. Black circles = G/G homozygotes; grey triangles = A-carriers. Lines constitute the linear models for G/G homozygotes (black) and A-carriers (grey). *r*-values refer to Pearson’s r correlations. (**B**) Comparison between the number of Instagram posts in G/G homozygotes (black) and A-carriers (grey) divided into high and low paternal care (* *p* < 0.025).

A significant interaction between paternal care and genotype also emerged for the number of Instagram posts (β = −0.03, SE = 0.01, *t* = −3.60, *p*= 0.0008). The distribution of genotypes, GG vs. A-carriers was not significantly different between high vs. low paternal care ($X^{2}$ (1) = 0.61, ns). No main effect of genotype or other interactions with genotype were significant. Paternal care was positively associated with the number of Instagram posts for A-carriers (*t*(24) = 4.27, df = 22, *r* = 0.67, *p*< 0.0003), but negatively associated with the number of Instagram posts for G/G homozygotes (*t*(33) = −1.42, df = 31, *r* = −0.25, ns) (see Figure 2). Although only one Pearson’s *r* was significant, the difference between the slopes for G/G and A-carriers, calculated with Fisher’s *z*, was statistically significant (*z* = 3.76, *p*= 0.0002). As predicted, the one tailed post-hoc Student’s *t* tests on the A-carriers vs. G/G in the low and high paternal care groups (corrected α=0.025) revealed that the number of Instagram posts was significantly different between A-carriers and G/G homozygotes only when they reported past experiences of low paternal care (*t* = −2.35, df = 29, *p*= 0.013, *d* = 0.85), but not when they had past experiences of high paternal care (*t* = 1.91, df = 24, *p*= 0.034, *d* = 0.79) (see Table 5 and Figure 2). The homogeneity of variance of the number of Instagram posts by paternal care was verified ($K^{2}$(1) = 0.49, ns).

#### 3.2.2. Social Desirability Index

The results of the multiple regression on the standardized SDI reported a significant main effect of maternal overprotection and a significant interaction between the same covariate and the genotype ($R^{2}$ (95% CI [0.00, 0.36]) = 0.22, power = 0.77). Although not the main focus of the paper, a main effect of maternal overprotection was found for the SDI (β = −0.03, SE = 0.01, *t* = −2.58, *p*= 0.013). However, post-hoc two tailed Student’s *t*-test revealed that the SDI was not significantly different between the low and high maternal overprotection groups (*t* = 0.50, df = 55, ns) (see Table 5).

**Figure 3:**
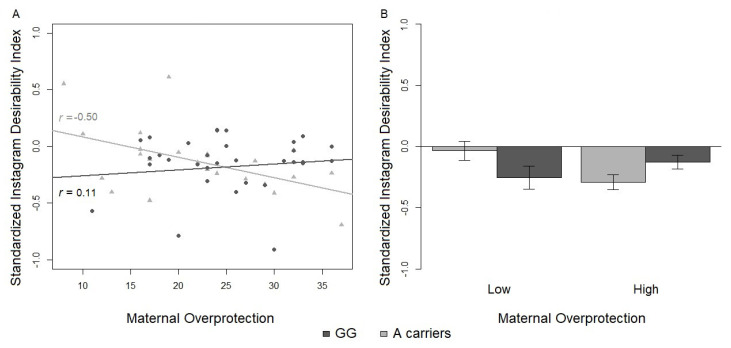
(**A**) Effect of the interaction between maternal overprotection and genotype on the standardized Instagram Social Desirability Index. Correlations between the Social Desirability Index and the reported maternal overprotection. Black circles = G/G homozygotes; grey triangles = A-carriers. Lines constitute the linear models for G/G homozygotes (black) and A-carriers (grey). *r*-values refer to Pearson’s r correlations. (**B**) Comparison between the Social Desirability Index in G/G homozygotes (black) and A-carriers (grey) divided into high and low maternal overprotection.

From exploratory analyses, a significant interaction between maternal overprotection and genotype emerged for the Instagram SDI (β=0.04, SE = 0.02, *t* = 2.78, *p*< 0.008). The distribution of genotypes, GG versus A-carriers, was not significantly different between high and low maternal overprotection ($X^{2}$ (1) = 1.51, ns). No main effect of genotype or other interactions with genotype were significant. Maternal overprotection was negatively associated with the Instagram SDI for A-carriers (*t*(24) = −2.72, df = 22, *r* = −0.50, *p*= 0.0125), but positively associated with the Instagram SDI for G/G homozygous (*t*(33) = 0.61, df = 31, *r* = 0.11, ns) (Figure 3). Although only one Pearson’s *r* was significant, the difference between the slopes for G/G and A-carriers, calculated with Fisher’s *z*, was statistically significant (*z* = −2.32, *p*= 0.02). Moreover, one tailed post-hoc Student’s *t* tests on the A-carriers versus G/G in low and high maternal overprotection groups (corrected α=0.017) revealed that the SDI was not significantly different between A-carriers and G/G homozygotes when they reported a past of low maternal overprotection (*t* = 1.79, df = 27, *p*= 0.04, *d* = 0.67), as well as a past of high maternal overprotection (*t* = −1.79, df = 26, *p*= 0.04, *d* = 0.73) (Figure 3). No significant difference was found on A-carriers between low and high maternal overprotection (*t* = 2.32, df = 22, *p*= 0.03, *d* = 0.98) (Table 5). The homogeneity of variance of the SDI by maternal overprotection was verified ($K^{2}$(1) = 3.16, ns).

#### 3.2.3. Number of Instagram Followings and Followers

No significant main effects of the covariate, main effect of genotype, or interactions with genotype were found.

### 3.3. Instagram Effects: OXTr rs53576

No significant main effects of the covariate or genotype were identified. Contrary to the proposed hypothesis, no significant interactions between maternal care, maternal overprotection, paternal care, or paternal overprotection and OXTr rs53576 were found.

## 4. Discussion

This study investigated how alleles in OXTr rs2254298 and rs53576 interact differently with parental care and overprotection during childhood in explaining Instagram social behavior. Two genotype*environment interactions (OXTr rs2254298 SNP * parental bonding in childhood; OXTr rs53576 SNP * parental bonding in childhood) on the number of Instagram followings and posts were hypothesized.

In line with the hypotheses in this study, adult Instagram users with a genetic risk factor (OXTr rs2254298 G/A or A/A genotype) show differential Instagram social activity according to their own early experience with parents. In particular, A-carriers, when exposed to a less optimal early environment (represented by low scores in parental care), showed a lower number of Instagram posts compared to less vulnerable genetic carriers (G/G homozygotes). Interestingly, A-carriers with a reported history of maternal overprotection also showed a decreasing trend in the Instagram SDI, whereas those with low scores in maternal overprotection showed an increasing trend in the same index. Overall, A-carriers who experienced negative patterns of interactions with their caregiver in childhood, as indicated by low paternal care and high maternal overprotection, exhibited weakened social responses on Instagram. From an analytical view, both interaction effects were found to survive the magnitude of the Bonferroni correction, which was differentially applied for the predicted (i.e., on the number of Instagram posts) and exploratory analysis (i.e., on the Instagram SDI).

To the authors’ knowledge, this is the first study that analyzes Instagram activity from a gene-environment perspective. Specifically, the present research examines Instagram behavior, a novel area to be explored, on a non-Western sample, which is usually under-represented in psychological studies [90]. Furthermore, these results corroborate the literature, which highlights the interaction effects between genetic predispositions and early social behaviors on human development. The moderating impact of genetics on environmental effects over Instagram social activity underpins the sensitivity hypotheses [91]. Within this framework, researchers have asserted that humans less susceptible to the environment (OXTr rs2254298 G/G homozygotes) will be less affected by stressful and negative conditions, but also by calming and positive events [44,61]. In contrast, in terms of conditional adaptation, humans with higher biological innate environmental susceptibility (OXTr rs2254298 A-carriers) display two dissociated behavioral patterns in relation to the quality of their early caring experiences and the consequent psychosocial outcomes [46,56,92,93]: (a) if exposed to a maladaptive environment and met with strenuous adversities, these individuals would show poorer sociable attitudes towards conspecifics compared to less susceptible individuals; (b) if exposed to an adaptive environment and functional experiences, these individuals would show higher functional social characteristics in the course of development and would cope better with stressful events compared to less sensitive individuals [45,48,94,95].

In connection with the present findings, the G allele predisposes users to be less vulnerable, but also less plastic to the environment, thus displaying a relatively fixed Instagram social pattern less associated with the quality of early parental caregiving; whereas the A allele increases the vulnerability to the environment and moderates online social behavior: (a) individuals with low paternal care are less social users (in terms of low number of posts) than G/G homozygotes on Instagram; (b) individuals with low maternal overprotection show an opposite trend of general sociability (in terms of high SDI) compared to G/G homozygotes on Instagram. However, it is important to specify the parameters from which a differential level of sociability as a function of the two cross gene*environment interactions can be inferred.

Firstly, A-carriers with low paternal care posted less than G/G homozygotes. A plausible argument is that lower paternal care could have determined decreased abilities in coping with Instagram’s overstimulating and socially demanding environment. In turn, less adaptive online behaviors could be explained by users’: (a) reluctance to be a proactive agent of the social network; (b) reluctance to be exposed to others’ judgment or influence [81,96]. An alternative explanation could be that these users are not interested in self-promotion or the development of social ties by posting [97].

Secondly, A-carriers with high levels of maternal overprotection showed a decreasing trend in the Instagram SDI, whereas the opposite pattern was observed in A-carriers with lower levels of maternal overprotection. The SDI was calculated to estimate the asymmetry between the number of followers and followings for each Instagram account. Here, a history of high maternal overprotection, potentially linked to the repression of child’s emotional expressions and autonomy [98], could shape the structure of the Instagram user’s social network: the higher the SDI, the higher the number of followers at the expense of the number of followings, the higher the recognition that each user receives from other Instagrammers. This being said, a higher grade of social desirability could be more likely achieved with increased self-management [99], more intense online activity, and the ability to attend to followers’ requests or expectations [28], thus all attitudes that could be undermined by high levels of maternal overprotection experienced in childhood [100,101]. This index could be even more useful in clustering different groups of users and distributing the scoring along a continuum from a minimum to a maximum number of followers over followings. Users who are not skilled with Instagram services will show a limited number of followers, while more competent users with good quality content will get higher follow back rates. Interestingly, Instagram posting activity and the SDI results could be strongly linked parameters since the former could characterize the first means to enhance the latter.

To conclude, Instagram users were affected by their reported paternal bonding, as well as maternal bonding. The detection of interaction effects that included one dimension for each parent provides a more balanced representation of the potential contribution of the parents in caregiving behavior.

From an educational perspective, a relationship promoting dialogue between parents and kids could represent a valuable resource in guiding social behavior towards peers [102] and supervising Internet and social media usage [103]. A trusting, but not excessively controlling presence of the parents may allow increasing the autonomy of the child in managing social media content [104]. A moderating action of parents in the administration of online life (i.e., defining a maximum duration of use, watching digital content with parents, discussing and clarifying the visualized content with parents) can train their child’s ability to recognize the potential risks of the web [105] and reduce the risks of developing addiction associated with the problematic use of the Internet and online services (i.e., Internet use disorder, Internet gaming disorder, Hikikomori syndrome) [106,107,108], such as social media [109,110].

Nevertheless, no significant interaction effects between OXTr rs53576 and parental bonding in childhood on Instagram social behaviors were detected. This result prompted us to elaborate on the following reasons. At a pragmatic level, different from rs2254298, the distribution in the sample for rs53576 A/A homozygous and G-carriers did not find total correspondence with the range of averaged distributions of these genotypes in the general Asiatic population. There could be indeed a remarkable variability in the alleles’ frequency in the Asiatic population, for example between East and South Asia (1000 Genomes project, BioSamples: SAMN07486027-SAMN07486024, dbSNP (Short Genetic Variations), 2017). Concerning this, it is arduous to ascertain the genetic distribution in a country like Singapore. At a more theoretical level, although there are several pieces of evidence of correlations between rs53576 polymorphism and pro-social tendencies and empathy [65,111], rs53576 could be more related to other explicit forms of sociality than online sociality itself. This statement could find support from two meta-analyses [66,112], which found a lack of association between rs53576 and general sociality. These considerations, taken together, could point to further investigation on the specific functionality of the single-nucleotide polymorphism in the region of rs53576.

Good caregiving practices could boost social approach, even in SNSs. In light of these considerations, oxytocin receptor gene polymorphisms (rs2254298) and early caregiving behaviors contribute to the modulation of Instagram user behavior. These findings shed light on a specific side of social behavior: an online social marker of the interaction between genetic and environmental factors.

## 5. Limitations and Future Directions

The current study has several limitations. First of all, the sample size was determined by study constraints and had a prevalence of the female gender. In the last decade, genetic association studies with limited sample sizes have become the target of increasing concerns related to the small variance explained in variables by one single-nucleotide polymorphisms (i.e., OXTr rs53576) [113,114]. However, given the difficulty of unraveling the nature of a given psychological construct (i.e., online social behavior) in a gene-environment perspective, the same experts of the fields cannot provide a definitive and unique solution to this implicit limitation [113]. For instance, the only evidence that oxytocin is a neuropeptide highly involved in a variety of functions [115] and expressed in several brain regions could exponentially boost the probability of detecting a significant effect [116]. A large number of loci could explain a given phenotype [117]. Here, only the genetic candidates OXTr rs2254298 and rs53576 were selected with care based on their well-documented functional properties [67], the hypothesis formulated for this study, and the results from previous works in the field [50,62]. From a statistical viewpoint, Cohen’s rule of interpreting the magnitude of a significant result [118] gave evidence that the significant effects (i.e., the interaction between paternal care and OXTr rs2254298 over Instagram posts number; the interaction between maternal overprotection and OXTr rs2254298 over the Social Desirability Index) were medium. The effect sizes were also consistent with the post-hoc power estimation. Other studies of behavioral genetics found comparable post-hoc statistical power in a small sample size [50,62]. Despite the efforts in data collection and genotyping, the current results should be interpreted with caution in light of the current debate on genetic association studies. Although it was not possible to maximize the sample size, a conservative approach to data treatment was adopted, and the results were in line with the expected patterns and defined by a reliable statistical magnitude. This study, in an exploratory way, deals with gene-environment interactions applied to new and recent variables from social media (i.e., Instagram social behavior), thus paving the way for further studies on this novel phenomenon.

Concerning the second limitation, no significant interaction effects were found for OXTr rs53576, presumably due to an unbalanced distribution in allele frequency within the sample. Further investigation could focus more on the role of OXTr rs53576 as a potential moderator of online social dispositions on SNSs such as Instagram. Third, the self-reported PBI questionnaire provides a retrospective measure of the individual self-perception of attachment, which could lead to influential biases. Alternative paradigms could include: (a) observational techniques as a more direct measure of caregiving patterns in an ecological context; (b) a longitudinal approach able to collect information on parental practices from childhood to adolescence. Fourth, four a priori objective parameters that were able to provide a reliable measure of each participant’s Instagram behavior were selected. Subsequent research could adopt different variables or indexes to explore the variegated world of Instagram. Overall, future studies should consider advanced interactions between new genetic factors (i.e., genetic polymorphisms of serotonin, dopamine) and environmental factors (i.e., membership within peer groups, socioeconomic status), but also hormonal components (i.e., menstrual stages in women, testosterone levels in men) and social characteristics (i.e., adult attachment with partners, quality of adult relationships) and individual traits (i.e., personality, education). All together, these variables could grasp common and specific mechanisms that characterize offline versus online social behavior.

## Figures and Tables

**Table 1:** Summary of Cronbach’s α for each subscale of the Parental Bonding Instrument.

PBI Subscale	Lower α	Raw α	Upper α
Paternal care	0.83	0.88	0.93
Maternal care	0.88	0.91	0.95
Paternal Overprotection	0.79	0.85	0.91
Maternal Overprotection	0.84	0.89	0.93

**Table 2:** Summary of the descriptive statistics for each continuous variable. The distribution of each variable is described in terms of the Minimum (Min), first Quartile (1st Q), median, mean, third Quartile (3rd Q), and Maximum values (Max).

Variables	Min	1st Q	Median	Mean	3rd Q	Max
Followings Number	−1.54	−0.77	−0.12	−0.12	0.52	1.80
Posts Number	0.33	0.39	0.49	0.56	0.67	1.19
Followers Number	−0.80	−0.50	−0.16	−0.11	0.22	1.45
Social Desirability Index	−1.16	−0.28	−0.13	−0.16	−0.03	0.61
Maternal care	0.00	4.00	8.00	9.97	14.00	28.00
Paternal care	0.00	9.00	14.00	14.49	18.00	30.00
Maternal overprotection	8.00	18.00	23.00	23.82	30.00	37.00
Paternal overprotection	11.00	23.00	29.00	27.05	32.00	37.00

**Table 3:** Summary of skewness and kurtosis values for each Instagram variable. The log-transformed number of posts shows an enhancement of the power values compared to the same non-adjusted variable.

Variables	Skewness	Kurtosis
Followings Number	0.29	−0.72
Posts Number	1.69	2.56
Log-transformed Posts Number	1.22	0.82
Followers Number	0.79	0.17
Social Desirability Index	−0.70	2.63

**Table 4:** Mean values in male and female participants on the overall Instagram variables. Standard Error Means (SEM) are reported between parentheses.

Instagram Variables	Males	Females
Followings Number	0.32 (0.23)	−0.28 (0.12)
Posts Number	0.60 (0.05)	0.55 (0.03)
Followers Number	0.08 (0.12)	−0.19 (0.08)
Social Desirability Index	−0.11 (0.03)	−0.18 (0.05)

**Table 5:** Top section: mean values of variables with main effects. Line 1: mean values in low and high paternal care on the number of posts. Line 2: mean values in low and high maternal overprotection on the Social Desirability Index. Bottom section: mean values of significant interactions. Line 1: mean values in A-carriers and G/G homozygotes divided into low and high paternal care on the number of posts. Line 2: mean values in A-carriers and G/G homozygotes divided into low and high maternal overprotection on the Social Desirability Index. Standard Error Means (SEM) are reported between parentheses.

**Instagram Variables**	**Low**	**High**		
Posts Number	0.54 (0.04)	0.59 (0.05)		
Social Desirability Index	−0.14 (0.06)	−0.18 (0.05)		
**Instagram Variables**	**Low/A**	**Low/GG**	**High/A**	**High/GG**
Posts Number	0.46 (0.03)	0.62 (0.06)	0.70 (0.10)	0.52 (0.05)
Social Desirability Index	−0.04 (0.08)	−0.25 (0.10)	−0.29 (0.06)	−0.13 (0.06)

## Supplementary Materials

The following are available online at https://www.mdpi.com/1660-4601/17/19/7232/s1, Table S1: Parental bonding styles, Table S2: Summary of Bartlett tests, Table S3: Summary of variance inflation factors.

## Abbreviations

The following abbreviations are used in this manuscript:

SNSs	Social Network Sites
OXTr	Oxytocin receptor gene
PBI	Parental Bonding Instrument
SNP	Single-Nucleotide Polymorphism
SDI	Social Desirability Index

## Appendix A. Pre-Registration

The public registration of this study can be found on the Open Science Framework at the following address: https://osf.io/j9nqc.

## Appendix B. Data Repository

The data of this study can be found in the NTU’sData Repository (DR-NTU Data) at the following address: https://doi.org/10.21979/N9/ZEH2XC.

## Appendix C. Ethics

The research was approved by the Ethical Committee of Nanyang Technological University (IRB-2015-08-020-01). Informed consent was obtained from all participants, and the study was conducted following the Declaration of Helsinki. The genetic assessment was conducted on anonymized bio-samples at the Nanyang Technological University (Singapore). Instagram behavioral data and questionnaires’ data were anonymized at the beginning of the data collection.
